# Exploring medicines management by COPD patients and their social networks after hospital discharge

**DOI:** 10.1007/s11096-018-0688-7

**Published:** 2018-07-28

**Authors:** Ellen Ingrid Schafheutle, Tom Fegan, Darren M. Ashcroft

**Affiliations:** Division of Pharmacy and Optometry, School of Health Sciences, Faculty of Biology, Medicine and Health, The University of Manchester, Manchester, UK

**Keywords:** Chronic obstructive pulmonary disease (COPD), Health networks, Medication work, Patient perspective, Qualitative interviews, Social networks, United Kingdom

## Abstract

*Background* Unplanned hospital admissions (UHAs) for chronic obstructive pulmonary disease (COPD) are a major burden on health services. Effective medicines management is crucial to avoid such admissions but little is known about the role of social networks in supporting medicines-taking. *Objective* To examine the activities and strategies recently discharged COPD patients and their social network members (SNMs) utilise to manage their medicines. *Setting* COPD patients recently discharged from an acute NHS Trust in Northwest England. *Methods* Semi-structured, face-to-face interviews; audio-recorded and transcribed with consent, NVivo v11 facilitated qualitative thematic analysis. NHS ethical approved. *Main outcome measure* Interview topic guide and analysis informed by Cheraghi-Sohi et al.’s conceptual framework for ‘medication work’ exploring medication–articulation, informational, emotional and surveillance work. *Results* Twelve interviews were conducted during March–August 2016. Participants’ social networks were small (n < 5) and restricted to family members and healthcare professionals. Participants social network members performed similar medication–articulation and surveillance work to coronary heart disease, arthritis and diabetes patients. When participants social network members resolved issues identified by surveillance work, this medication work was conceptualised as surveillance–articulation work. The social network members performed little emotional work and were infrequently involved in informational work despite some participants describing informational needs. After discharge, participants reverted to pre-admission routines/habits/strategies for obtaining medication supplies, organising medicines, keeping track of supplies, ensuring adherence within daily regimens, and monitoring symptoms, which could cause issues. *Conclusion* This study applied Cheraghi-Sohi’s framework for medication work to COPD patients and described the role of the social network members. Pharmacists could proactively explore medication infrastructures and work with patients and their close social network members to support medication work.

## Impacts on Practice

- The social networks of COPD patients who were discharged from hospital seem small but—where involved—played significant roles in medication articulation and surveillance work.
- Health care professionals are mainly involved in supporting medicines articulation work through supply and monitored dosage systems (MDS) information needs were rarely articulated.
- Pharmacists could target COPD patients following discharge, to identify their social networks and help patients with their social network members establish or amend medication infrastructure; they could also be proactive and support informational work.

## Introduction

Worldwide, an estimated 384 million people are affected by chronic obstructive pulmonary disease (COPD), and its global prevalence is increasing [1]. In the UK, about one million people have been diagnosed with COPD [2] with 30,000 people dying each year as a result of COPD [3] being the fourth leading cause of mortality in England and Wales [4, 5]. In England, COPD is the second most common cause of emergency admission to hospital and around a third of patients are readmitted within a month of discharge [5].

COPD is a progressive chronic respiratory disease with patients typically suffering from shortness of breath, tight chest, chronic cough and chronic sputum production [6]. Symptoms can vary within and between days [7–10] and COPD affects people’s ability to perform daily activities and can affect sleep [11]. Those with severe COPD may be house-/chair-bound and socially isolated [12]. The high symptom burden progressively impacts on patients’ quality of life by reducing physical and social functioning [12].

Medicines reduce symptoms, frequency/severity of exacerbations and improve health status [6, 13]. Patients with COPD may be prescribed multiple medicines and formulations (e.g. tablets/capsules, inhaler devices and nebulisers) [6, 13, 14]. As 80% of COPD patients have at least one additional co-morbidity [15], which are also treated with prescribed medicines, the burden of treatment is large [16, 17]. Furthermore, exacerbations are typical for COPD patients and commonly lead to a change in medication and/or the use of rescue therapy [6]. The complexity of COPD medication regimens is a known cause of unintentional non-adherence [18–22].

The concept of self-care has developed to recognise the role of patients’ social networks, and an increasing number of studies are exploring the structure and function of patients’ social networks in chronic illness management [23–27]. Recently, Cheraghi-Sohi et al. [28] explored the role of ‘health networks,’ i.e. social network members seen important to managing their health condition(s), in helping patients with diabetes, coronary heart disease (CHD) and arthritis to take their medicines and identified four types of ‘medication work:’ medication–articulation, informational, emotional and surveillance work. The most frequent type of work, *medication*–*articulation*, includes the act of taking or administering medicines and the activities performed to maintain adequate stock and facilitate medicine-taking at instructed dose times. *Surveillance work* involves monitoring supplies of medicines to ensure adequate personal stock, mitigating against dispensing errors, and keeping track of medicine-taking within daily regimens. *Emotional work* involves reassurance, reciprocation and prompting medicine-taking, and helps improve adherence to medicines. Finally, *informational work* includes clarification and checking information received from other network members and resolving medication concerns.

Given the economic and health cost of COPD, particularly related to hospitalisation, and the high treatment burden [16, 17], it is important to understand the work involved for COPD patients and their social networks to take their medicines after discharge from hospital.

## Aim of the study

The aim of the study was to describe the activities/strategies patients with COPD utilise after leaving hospital to take their medicines and identify the social network members involved in these activities/strategies.

## Ethics approval

NHS research ethics committee (reference 16/WM/0039) and research and development approvals were obtained.

## Methods

A qualitative approach was chosen to enable the attitudes, behaviours, views, beliefs and experiences of individuals to be explored in-depth [29]. Cheraghi-Sohi et al.’s [28] concept of ‘medication work’ was used as a theoretical framework, and semi-structured interviews were conducted to explore participants’ social network members and medicines-taking behaviours [30]. These were conducted face-to-face in participants’ homes, taking account of physical restriction and social isolation associated with COPD [31]. Where participants wished, joint interviewing (with social network members) was supported [32], as it allowed enriched accounts due to participants interacting with their social network members [33, 34].

The initial topic guide (Table 1) [28], asked questions about a variety of medication related topics and the social network members involved in patients’ daily medication routine, including ordering and collecting medications, maintaining supply, and organisation of medications around the home. Network diagrams were used to identify participants’ social network members and their relative importance to medication work. Conducting interviews in participants’ homes allowed observation of their ‘social world,’ reminding them about their routines [35], which gave a sense of the spatial components of medication work systems, such as where medicines were stored/located.

**Table 1:** Topic guide—main themes/questions

*Background section* Number of long-term conditions, time of COPD diagnosis, medicines and recent changes, where medicines are kept
*Network section* Explain social network diagram [prompt: family, friends, neighbours, HCPs, pets] Social Network Members
Explore how getting on with taking medicines since returning home Describe typical day in terms of medicines taking How remembering when to take medicines Activities that help/get in the way People that help/get in the way How convenient are each of your medicines to take? # prompt: individual meds; meals; co-ordination; fridge; rinsing mouth after steroids; rescue meds; prn therapy; oral steroid as single dose in the morning after breakfast; palatability; swallowing; side effects How know the taken all medicines # prompt: reminders e.g. people, diaries, record-keeping, blister packs, meals, symptoms, routine/habit [if applicable] Have new medicines affected medicine-taking routine/habits? How? Have you had enough of the medication that you have needed? Encountered any problems obtaining your medicines? How managed these? People: who? [refer to network diagram] Activities: what? Visited GP, pharmacist or nurse about medicines [refer to network diagram]—what for? Medicines? Faced any challenges taking medicines Done anything differently to help with taking medicines since returned home? Anything else to say about what, or who, has helped you to take your medicines since returned home?

### Sampling

Sampling was purposive for gender, severity of disease and discharge pathway (but not social network typologies [25] which were unknown), with maximum variation to increase heterogeneity of the sample and the range of accounts generated [29]. Adult patients, admitted to and discharged from an acute National Health Service (NHS) Trust between March and August 2016, related to COPD, were eligible except those lacking capacity or a strong verbal command of English. All grades of disease severity and co-morbidities were included and participants were recruited from two discharge pathways: inpatient respiratory wards and ‘Hospital at Home’ scheme, which allows earlier discharge from the assessment ward with additional support from the nursing team. Clinicians identified eligible patients and provided brief verbal and written information. Patients were asked to indicate in writing their consent to be contacted by the researcher, who followed up patients within a week.

### Data analysis

Interviews were conducted by one of the authors (TF), audio-recorded and transcribed verbatim with written consent. ‘Field notes’ were made immediately after the interview to capture the researcher’s “impressions and perceptions of emergent issues and feelings” [36], which served as a latent, theoretical thematic analysis [37].

Transcripts were coded in NVivo 11, with each transcript acting to check and develop the coding framework; memos were made and compared with field notes. A (non-linear) “recursive process” [37] enabled coding to be reworked and developed into themes. Once all the transcripts had been coded, data extracts were interrogated by themes. While analysis of data fitting Cheraghi-Sohi et al.’s [28] conceptual framework was deductive, theoretical and analyst-driven, the analysis was inductive for data that did not fit the framework [38]. Sampling, data generation and data analysis continued until saturation was reached. While TF led the coding and analysis, he did so in close collaboration with his two co-authors (ES, DA) who are experienced health services researchers, with ES having particular expertise in qualitative research. At the end of the study, a brief lay summary of findings was distributed to all participants, for information.

## Results

Forty-three patients consented to be contacted and 12 participants (six from each discharge pathway) agreed to interviews, which were conducted between March and August 2016, lasting between 25 and 59 min (average 39 min). Seven participants were males; participants were aged between 56 and 84 years. Network typologies ranged from isolates with no personal social network members to small networks consisting of no more than five social network members. Most participants identified spouses as their most important social network members, adult–children and grandchildren also played an important role for some; pharmacies had some importance for two participants, but otherwise healthcare professionals featured rarely as social network members (see Table 2). Disease severity ranged from new COPD diagnosis to history of regular hospital admissions; the number of long-term conditions ranged from single COPD diagnosis to multi-morbidities.

**Table 2:** Summary of sample demographics and social network members (SNMs)

Participant	Gender	Age	SNM present during interview	Most important^a^ SNM (inner ring)	Important^a^ SNM (middle ring)	Least important^a^ SNM (outer ring)
A	Female	70		Adult grandchild	–	–
B	Male	56		Spouse/partner	Adult–child	–
C	Female	84		–	–	–
D	Male	72		Pharmacy	Spouse/partner	–
E	Female	68		Spouse/partner	–	–
F	Male	64	Wife	Spouse/partner	–	Pharmacy
G	Female	59	Family carer	*	*	*
H	Male	67		Spouse/partner	Pharmacy	Adult–child/healthcare professional
I	Male	75	Wife	–	–	–
J	Male	69		–	–	–
K	Female	81	Family carer (daughter)	*	*	*
L	Male	69		Spouse/partner	–	Adult child

*Participants were not asked to complete network diagrams

^a^SNMs marked in the inner ring of network diagrams were those considered most important to medication work by participants; SNMs marked in the outer ring were the least important to participants

Findings are presented using Cheraghi-Sohi et al.’s framework [28]. The most frequent type of medication work performed by participants and social network members was medication–articulation work.

### Medication–articulation work

#### Organisation work

For participants taking larger numbers of medicines, organising medicines was a considerable part of medication–articulation work. Participants utilised different and personalised strategies, whereby participants or social network members stored original packs in cabinets/cupboards or a shoe box in the kitchen and took medicines directly from original packs.

Many participants repackaged their monthly supplies into shorter time periods. Some participants used clear plastic bags or medication bottles; others used pharmacy-assembled monitored dosage systems (MDSs). A few participants and/or social network members assembled their own pill boxes, which took 1–2 h each month. These repackaging strategies reduced administration work by reducing the time spent taking medicines at each dose time.

Me and [my partner] just spend an hour on the table in there [kitchen/dining room] and just stick them all on the table and sort them all out as we need them (participant D).

The use of monitored dosage systems (MDSs) delegated organisation work to pharmacies while pill boxes and other strategies delegated this work to immediate family members. One participant’s carer did not instruct the pharmacy to assemble MDS due to a fear of potential dispensing errors and/or supply problems, even though organising her mother’s medicines demanded significant time.

#### Location, location, location

Medication–articulation work also included organising medicines in and around their homes. A number of participants placed individual or a day’s worth of doses in visible and frequently visited locations around the home to visually prompt them to take medicines at dose times. This form of medication–articulation work underpinned the surveillance work performed to keep track of progress within daily regimens.

I mean I’ll leave [the next dose of medicines] in front of me I know then if forgotten (participant A).

The placement of fast-acting inhalation therapies within easy reach of participants’ sitting positions during interviews appeared deliberate, such co-location being another form of organisation work. While some participants could not leave the house after discharge due to their illness, a small number described strategies to ensure medicines were also co-located when leaving the house.

It’s also one of those (pill boxes) where you can flip them out and take a stick of four out and take them with you anywhere if you’re going for a day out (participant D).

I have a portable nebuliser, battery driven, so I can take my nebs, that’s not a problem. […] there’s a portable nebuliser in the car (participant L).

Some participants placed medication in specific locations to protect immediate family members.

The oral tablets are kept in a dosette pack, which is provided by the pharmacist, and I just keep those in the kitchen at hand, well, out of reach of children (participant H).

#### Administration work

Taking oral and inhaled medicines was performed by participants placing multiple tablets in paper cups/hands before taking them at once or one tablet at a time.

I just drop them all in [a little round tub], count them, make sure I’ve got them all and then just open it up and just drop them in my hand, a few at a time and then I have a cup of tea (participant B).

Many participants found the physical act of taking medicines unproblematic; some participants described swallowing difficulties due to tablet sizes, palatability and dexterity issues, and vomiting, which was particularly burdensome for participants taking higher numbers of medicines. Participants described strategies such as taking medicines with yoghurts and drinks, and swallowing multiple pills simultaneously, to overcome these difficulties.

It’s the amount of tablets and the size of them. I’m finding it hard to swallow at the moment (participant K).

Some described how social network members became involved in administration work after discharge by helping participants with newly prescribed inhaler devices and placing medicines in paper cups.

#### Ordering and obtaining medicines

Medication–articulation work also included the ordering, collection and delivery of medicines, which ensured constant medication supply. While most participants ordered their medicines themselves, collection was mostly performed by immediate family members and delivery by pharmacies. Knowing when to request a new prescription was a form of surveillance work underpinning this medication–articulation work.

### Surveillance work

Participants described several types of surveillance work.

#### Ensuring a constant supply

Before a new supply of medication could be obtained, participants or pharmacies needed to contact GPs to request new prescriptions. Some participants described how low levels of stock and/or calendar dates, either stated on repeat prescription slips and/or ‘fixed’ in their mind, helped them obtain new supplies before running out.

Say I’m running out [of medicines] by Friday I’ll phone them Monday and then they bring it [medicines] usually Thursday, Friday morning. So I don’t run out (participant A).

We look at the date [on the repeat slip], and I have to have a diary all the time to write things in (Wife: participant F).

It was not possible, however, to use stock levels to reliably determine when to order prescriptions for inhaler devices; some participants re-ordered these items when stock of other medicines was low.

The purple inhaler, it told you how many doses there are. But the blue one, you just wait until it starts running out (participant F).

One pharmacy “automatically” requested repeat prescriptions, and also assembled and delivered monitored dosage systems (MDS), thereby performing this type of surveillance and medication–articulation work.

The dosette packs are delivered to me on a monthly basis. […] When I’m onto the last week, they’ll deliver again. I don’t have to remind them. That’s just automatically done (participant H).

#### Monitoring medication supplies

Participants did not describe actively monitoring medication supplies; however, some felt that after many years of use they would identify errors if the number and/or appearance of medicines was visibly different.

I don’t consciously check [the MDS], but I could tell immediately, because with doing it every day, you get used to seeing what you tip out in your hand (participant H).

After discharge, some participants and/or social network members identified prescribing/dispensing errors, particularly related to insufficient stock and changes to medication regimens made in hospital.

It’s been a bit of a mess with [participant’s wife] really, ‘cause they’d given me a letter when they sent me back home from hospital saying I had to keep taking this medicine, don’t take that, keep taking this. So [participant’s wife] went down to the doctor’s surgery, and they gave her this medicine, and three days later my other one had ran out, so we had to go back again, and then two days later something else had ran out, and we had to go back again (participant I).

When surveillance work identified these problems, participants and/or their social network members had to perform surveillance–articulation work to obtain sufficient supply of the correct medicines before running out.

#### Monitoring symptoms and side-effects

Surveillance work also included monitoring symptoms. Monitoring side-effects was another less frequently performed type of surveillance work. One participant’s family carer, for example, monitored potential side-effects of oral anticoagulant therapy by looking out for persistent bruising.

#### Memory, routine and habit

Most participants relied on memory, routine and habit developed over time to know which medicines to take and when. Keeping track of progress within daily regimens was therefore “automatic” for many and thus seen as minimally burdensome.

Well in morning I take them before I have any breakfast, well I have a biscuit while I take them but then I have my breakfast after and then in afternoon I just take them two [pills] around half two, three o’clock and then the others round eight, nine o’clock. […] I just do it, I’ve done it that long it’s just automatic (participant A).

[Taking medicines] is just a routine thing. The dosette pack is labelled, you know, tablets in the morning, tablets at lunchtime, tablets in the evening, so that’s just what I do (participant H).

Waking/sleeping, breakfast/dinner, drinks and clock time reinforced routines and assured participants they had taken the correct medicines at the correct times. Sleep disturbances, however, were disruptive; afternoon naps and difficulties maintaining regular sleep patterns interfered with some participants’ morning and midday doses. Personal social network members (e.g. family carers, dog) mitigated against this by waking some participants at dose times.

If I didn’t wake her up, I think that she could sleep all through, without taking medication and that. I have to wake her up to give her medication (Family carer: participant G).

Some participants described difficulties with incorporating newly prescribed medicines, particularly when dosage regimens did not correspond with established routines and strategies. For two participants, the addition of midday doses to their ‘morning and night’ routine led to non-adherence due to forgetfulness and conflict with the organisation in their pill box. For another, the change of routine caused by newly prescribed inhalers resulted in missed or delayed doses.

I’ve not got used to doing it yet, properly, because I’m used to getting up and taking my other medicines for 41 years, that was like a habit and now it’s another two to get used to (participant B).

Incorporating newly prescribed medicines did not always present problems for participants, especially when participants and/or personal social network members were involved in organising medicines for participants and/or reconciling changes.

### Surveillance–articulation work

Surveillance work created a need for participants to act, conceptualised as ‘surveillance–articulation’ work to recognise the inter-dependence between medication–articulation and surveillance work.

#### Responding to symptoms

Surveillance–articulation work included participants taking ‘as required’, fast-acting, inhaled therapies, antibiotics and/or oral steroids in response to symptoms such as breathlessness and signs of infection.

What I have realised over the last four or five months is that if you do keep an eye on her and you monitor Mum you can nip [an exacerbation] in the bud. If you look and you see…you recognise the signs, you can stop an exacerbation by giving Mum the medication and you know, keeping her calm and keeping her comfortable and making sure she’s pain free. That way, you know you’ve got more chance of staying at home, because we do manage to nip it in the bud a lot now (Family carer: participant K).

If I were to get an infection, the signal for me is, if I get a sore throat, then I would immediately start the rescue pack of antibiotics, and steroids (participant H).

Some participants contacted healthcare professionals to confirm the appropriateness of initiating rescue therapy for exacerbations.

#### Prescribing/dispensing errors resolution

When surveillance work identified prescribing/dispensing errors, some participants and/or social network members contacted GPs and/or pharmacies.

[Following discharge from hospital] they said they’d sent you home with a week’s blisters, it’s normally a week’s blister pack but when Mum came home on Wednesday they’d already used the Monday, Tuesday and they’d give her Wednesday morning […] and the pharmacist thought Mum was going to be alright until the Wednesday (Family carer: participant K).

There were two examples of pharmacists proactively contacting participants to ensure a constant medication supply.

### Informational work

Participants described performing limited amounts of informational work. While some participants were uncertain about changes to medication regimens and newly prescribed inhalation devices, they rarely initiated contact with personal social network members and healthcare professionals to address these informational needs after discharge. Some participants consulted patient information leaflets (PILs) to learn about side-effects and how to use new medicines and inhaler devices. Some felt the extensive side-effects listed in PILs and difficulties interpreting the information limited their usefulness; these participants preferred to contact healthcare professionals about medication concerns.

The dosage instructions on medication labels helped participants know when to take their new medicines, but they did not generally read these labels for information. While there were unmet information needs, others did not need information and therefore did not perform or involve social network members in informational work.

I’ve been on them that long, the only information I needed were when I first started (participant J).

### Emotional work

There were examples of co-habiting social network members in this study performing activities that Cheraghi-Sohi et al. described for emotional work, such as waking participants and giving verbal prompts as militating against intentional non-adherence or tending to act as the ‘rebelling self,’ reciprocation and loading inhaler devices at dose times [28]. However, it was unclear whether participants believed these actions were expressions of concern, care or support.

In the evening, at nine o’clock, [participant F] reminds me to take my medicine, because I’m on [drug Y], and so we help each other (wife: participant F).

## Discussion

This study applied Cheraghi-Sohi et al.’s framework [28] by exploring, in-depth, the medication work of COPD patients and their social network members. Social networks were small and restricted to immediate family members, but not all spouses/partners were involved. Healthcare professionals were mostly involved in enabling new supplies of medicines and were reactive to concerns raised by participants and/or social network members. The present study describes the strategies used to facilitate medicines-taking by COPD patients and provides evidence of the role of social networks in helping patients ‘self’-manage medicines. The present study extends Cheraghi-Sohi et al.’s framework to include a fifth type of medication work: surveillance–articulation, as well as developing the framework by describing the inter-dependence between different types of medication work.

### Strengths and limitations of results

The main strength of this study is the central focus on the activities and strategies that help COPD patients take medicines from a network perspective. This study provides an in-depth, ‘thick’ description of COPD patients’ experiences of taking medicines. Whilst the sample was small, it was consistent with samples in qualitative studies and analysis showed that data saturation, defined as “data adequacy” and a requirement to “collect data until no new information is obtained” [39], was reached. Patients were recruited from only two discharge pathways in one NHS trust, but purposive sampling ensured heterogeneity, with a range of accounts generated and triangulation used to increase credibility of findings [40]. By conducting interviews in participants’ homes, observation enabled verbal accounts to be compared with what was visible.

### Medication work

The ‘medication work’ performed by participants and/or social network members in this study showed similarities and differences with Cheraghi-Sohi et al.’s study [28]. Patients placed medicines in and around the home as a form of medication–articulation work, often in visible and frequently visited locations, which served as reminders. The observed close proximity of inhalation therapy is consistent with strategies to control the living environment in order to reduce exertion of everyday work and manage physical restriction [41, 42]. Fear-avoidance behaviours could also explain the close proximity of inhalation therapy specifically for breathlessness [43–48].

Organising medicines was a particularly large part of medication–articulation work for COPD patients taking larger numbers of medicine, which commonly involved social network members, and helped them keep track of doses, where habit and routine were fundamental, a finding which is consistent with existing evidence [49–52].

Several differences in medication–articulation and surveillance work existed for COPD patients. Monitoring prescribing/dispensing errors, for instance, was generally reactive in contrast to the proactive surveillance work described by Cheraghi-Sohi et al. [28]. Administration work was considerably greater for COPD patients, particularly those taking higher numbers of medicines, and social network members—where involved—played significant roles. In addition, the ‘work’ performed to act appropriately in response to symptoms was unreported by Cheraghi-Sohi et al. [28]. The limited emotional work described in this study is contradictory to both Cheraghi-Sohi et al. [28] and wider evidence on social network members’ emotional support for COPD patients [43, 47, 53–55].

Cheraghi-Sohi et al. found that patients contacted healthcare professionals primarily for newly prescribed or changed medicines [28]. Given the existing evidence regarding changes to medication regimens during hospital stays and related information seeking [56, 57], it was surprising that COPD participants, despite similar informational needs, did not generally involve healthcare professionals or personal social network members in informational work after discharge.

The present study identified a fifth type of work, recognising the inter-dependence between medication–articulation and surveillance work: surveillance–articulation work, which describes the work performed to resolve problems identified by surveillance work. Surveillance–articulation work was needed to allow participants to perform medication–articulation work, i.e. planning and co-ordination work around medicines-taking. This type of medication work incorporates the concept of self-efficacy, which recognises that a patient’s ability to self-manage is based on their confidence in given situations, and information and training provided by healthcare professionals able to increase self-efficacy [43, 54, 58–60].

The present study also developed Cheraghi-Sohi et al.’s [28] framework by describing the inter-dependability of different types of medication work; for example, when medication–articulation work was performed inadequately by pharmacists, patients and/or social network members performed surveillance work to ‘check’ medication–articulation work. The inter-dependability of medication–articulation and surveillance work is recognised by the concept of surveillance–articulation work.

Some social network members became particularly involved after discharge, by helping patients with a newly prescribed medicine, helping to organise medicines and/or performing surveillance work. The involvement of social network members specifically during the post-discharge period is consistent with previously reported increased involvement of social network members during episodic and chronic deterioration of COPD patients [46, 61].

### Social networks

The numbers of social network members in the social networks of participants in the present study were small, which supports the network sizes previously reported [62, 63]. Personal social network members were limited to immediate family members, and the social networks were predominantly family-focussed, which is narrower than the ‘family-’ and ‘friend-focussed’ network typology described by Morris et al. [25]. The stronger reliance on close family networks in this study may be due to the relative severity of COPD symptoms in general and particularly post-discharge, in comparison to other long-term conditions [11, 12].

Some social network members were routinely involved in medication work, especially the medication–articulation work of organising medicines around the house. Personal social network members were involved in surveillance and surveillance–articulation work to resolve identified problems with medication supplies. While GPs and pharmacists facilitated surveillance–articulation, they had limited roles and were often reactive to concerns raised by patients and social network members.

That social network members, particularly family members cohabiting with patients, had significant and possibly more involved roles than those patients involved in Cheraghi-Sohi et al.’s study [28] may be due to the types of patients involved in this study. COPD patients take significant numbers of medicines, and they tend to be relatively sick—particularly in this sample after discharge following an exacerbation. Therefore, social network members may play a particularly significant role in medication articulation and surveillance work.

The involvement of healthcare professionals was limited in all forms of medication work, and existing literature offers several possible explanations. Physical restriction and social isolation could have prevented interactions with healthcare professionals outside the home, thereby relying on remote contact or visits by healthcare professionals [43, 44, 46, 53, 55, 56, 64–66]. Efforts to preserve one’s self-image could also have explained the limited involvement of healthcare professionals in medication work [48, 53, 66–68]. There is some evidence that suggests patients perceive the role of pharmacists as limited to medicines supply [26, 69–71].

## Conclusion

Medicines taking work is complex and burdensome for patients and, in many cases, social network members, particularly close family members. Patients with long-term conditions impacting on their ability to function fully and independently, such as COPD, may have particular needs. Healthcare professionals, and particularly pharmacists, could be proactive and identify not just medication requirements but also the role of close social network members, and target this at a time of likely change and particular burden, such as following discharge from hospital. They can then play a role in establishing or amending existing medication infrastructures to support both patients and their close social network members in their medication work.

## Figures and Tables

**Figure:**
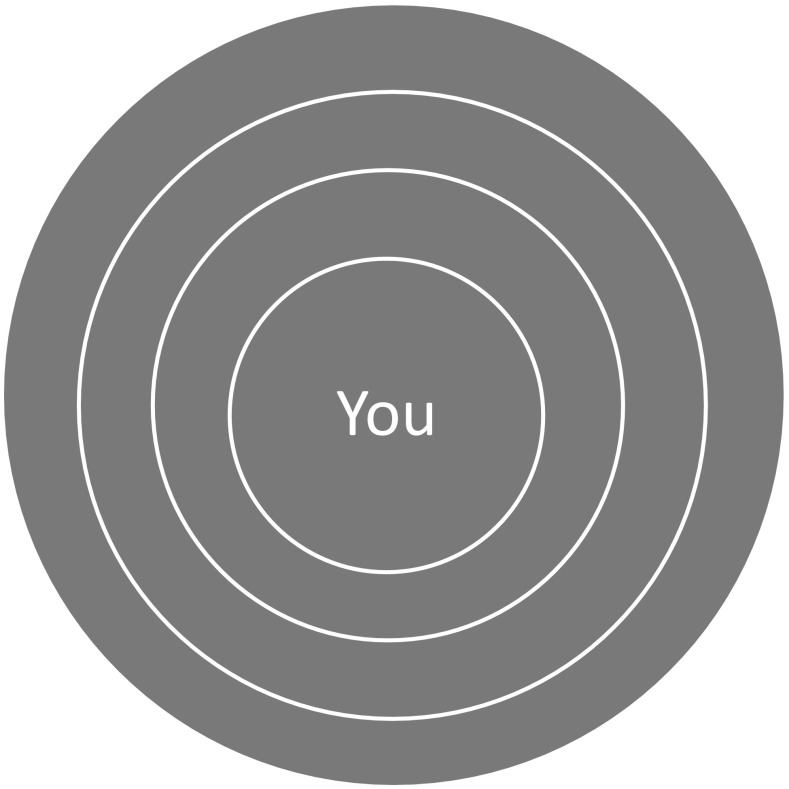
Self/ego (you) at centre, with closest social network members within *inner circle*, moving outwards to least important SNM (*outer ring*)
